# The association of circulating kynurenine, a tryptophan metabolite, with frailty in older adults

**DOI:** 10.18632/aging.104179

**Published:** 2020-11-13

**Authors:** Il-Young Jang, Jin Hoon Park, Jeoung Hee Kim, Seungjoo Lee, Eunju Lee, Jin Young Lee, So Jeong Park, Da Ae Kim, Mark W. Hamrick, Beom-Jun Kim

**Affiliations:** 1 Division of Geriatrics, Department of Internal Medicine, Asan Medical Center, University of Ulsan College of Medicine, Seoul, South Korea; 2 Department of Neurological Surgery, Asan Medical Center, University of Ulsan College of Medicine, Seoul, South Korea; 3 Asan Institute for Life Sciences, Asan Medical Center, University of Ulsan College of Medicine, Seoul, South Korea; 4 Department of Cell Biology and Anatomy, Medical College of Georgia, Augusta University, Augusta, GA 30912, USA; 5 Division of Endocrinology and Metabolism, Department of Internal Medicine, Asan Medical Center, University of Ulsan College of Medicine, Seoul, South Korea

**Keywords:** kynurenine, frailty, aging, sarcopenia, biomarker

## Abstract

Despite the accumulating evidence from *in vitro* and animal experiments supporting the role of kynurenine (a tryptophan metabolite) in a number of degenerative age-related changes, the relationship between kynurenine and frailty in older adults is not well understood. We collected blood samples from 73 participants who underwent a comprehensive geriatric assessment, measuring kynurenine levels using liquid chromatography-tandem mass spectrometry. We assessed the phenotypic frailty and the deficit accumulation frailty index using widely validated approaches proposed by Fried et al. and Rockwood et al., respectively. After adjusting for sex, age, and body mass index, the frail participants presented 52.9% and 34.3% higher serum kynurenine levels than those with robustness and prefrailty, respectively (*P* = 0.005 and 0.014, respectively). Serum kynurenine levels were positively associated with the frailty index, time to complete 5 chair stands, and patient health questionnaire-2 score and inversely associated with grip strength and gait speed (*P* = 0.042 to <0.001). Furthermore, the odds ratio per increase in serum kynurenine level for phenotypic frailty was approximately 2.62 (95% confidence interval = 1.22–5.65, *P* = 0.014). These data provide clinical evidence that circulating kynurenine might be a potential biomarker for assessing the risk of frailty in humans.

## INTRODUCTION

The public health burden of age-related diseases is increasing rapidly as the number of older adults continues to grow around the world. Hip fractures are a major cause of morbidity and mortality among the elderly, and approximately 40% of those who experience a hip fracture will end up in a nursing home, and 20% will never walk again. The 1-year mortality rate after hip fractures at 70 years of age is approximately 30%. Muscle weakness and postural instability are major contributors to the incidence of falls among the elderly, and falls are the primary etiological factor in 90% of hip fractures. Loss of muscle mass in the form of sarcopenia is estimated to affect approximately 30% of individuals over the age of 60 and more than half of individuals over the age of 80 [1]. Loss of muscle and bone mass with age is therefore a major limiting factor for life spans, and the morbidity that accompanies fractures in the elderly is costly both in terms of financial burden and quality of life. Moreover, reductions in lean mass and muscle strength also appear to precede cognitive decline, dementia, and Alzheimer’s disease [2–7]. The overall decline in muscle, bone and cognitive function that occurs with aging directly contributes to frailty, which is characterized by reduced physical performance, balance, muscle strength and endurance, and neuromuscular function.

Aging is associated with an overall increase in the inflammatory burden, driven at least in part by elevated local and circulating levels of inflammatory cytokines such as interleukin (IL)-6, IL-1β and interferon-γ. These inflammatory cytokines are known to stimulate the enzyme indoleamine 2,3-dioxygenase (IDO), which degrades the amino acid tryptophan along the kynurenine pathway (KP) [8, 9]. An increase in IDO activity has been associated with increased mortality in humans [10], and inhibition of tryptophan degradation, thereby reducing kynurenine accumulation, has been observed to increase longevity in animal model systems such as worms [11] and fruit flies [12]. Kynurenine has recently been shown to induce both muscle and bone loss in mice [13, 14], and kynurenine levels are elevated in patients with fragility hip fractures [15]. Importantly, inhibiting tryptophan degradation and IDO activity with the tryptophan mimetic 1-MT can improve muscle function in aged mice [14], suggesting that modulating kynurenine accumulation with aging might be a potential pathway for improving musculoskeletal function. Yet, despite several recent studies linking kynurenine levels to bone loss and fracture, there is a paucity of clinical data on the relationship between kynurenine and frailty in older adults. To determine whether circulating kynurenine could be a potential biomarker of frailty, we examined the kynurenine levels in a cohort of older adults whose frailty status differed significantly.

## RESULTS

### Clinical characteristics of the study participants according to phenotypic frailty status

Table 1 lists the baseline characteristics of the 73 study participants. Among 17 (23.3%) robust (i.e., non-frail), 44 (60.3%) prefrail, and 12 (16.4%) frail older adults based on Fried’s criteria [16], 8 (47.1%), 26 (59.1%), and 7 (58.3%) were women, respectively. The mean ages of the robust, prefrail, and frail groups were 67.6 ± 6.8, 69.8 ± 5.9, and 70.8 ± 5.0 years, respectively. There were no significant differences in terms of weight, height, body mass index (BMI), serum albumin level, time to complete 5 chair stands, and prevalence of polypharmacy and multimorbidity between the three groups. Compared with the robust and/or prefrail groups, the frail group had lower grip strength, gait speed, short physical performance battery (SPPB) score and mini-cognition score and higher frailty index, social frailty score and patient health questionnaire-2 (PHQ-2) score. The frail group was more likely to experience deficiencies in activities of daily living (ADL) and instrumental activities of daily living (IADL), as well as cognitive dysfunction and depression.

**Table 1:** Baseline characteristics of the study participants according to phenotypic frailty status.

**Variables**	**Robust (n = 17)**	**Prefrail (n = 44)**	**Frail (n = 12)**	***P***
Sex, n (%)				0.688
Female	8 (47.1)	26 (59.1)	7 (58.3)	
Male	9 (52.9)	18 (40.9)	5 (41.7)	
Age, years	67.6 ± 6.8	69.8 ± 5.9	70.8 ± 5.0	0.315
Weight, kg	71.3 ± 12.5	67.7 ± 10.4	63.3 ± 8.6	0.141
Height, cm	161.0 ± 11.0	158.8 ± 8.9	157.2 ± 5.6	0.522
Body mass index, kg/m^2^	27.5 ± 3.5	26.7 ± 3.0	25.7 ± 3.8	0.332
Serum albumin, g/dL	3.78 ± 0.38	3.85 ± 0.28	3.73 ± 0.25	0.418
Frailty index (range: 0–1)	**0.076 ± 0.035**	0.125 ± 0.061^*^	0.230 ± 0.083^*,†^	**<0.001**
Grip strength, kg	**31.7 ± 8.3**	**27.8 ± 9.1**	20.9 ± 6.6^*^	**0.007**
Gait speed, m/s	**1.20 ± 0.18**	**1.05 ± 0.33**	0.75 ± 0.28^*,†^	**0.001**
Chair stand, s	9.7 ± 4.6	11.0 ± 8.3	14.5 ± 7.2	0.243
SPPB score (range: 0–12)	**11.4 ± 1.1**	**10.7 ± 2.2**	9.5 ± 2.2^*^	**0.044**
Use of ≥5 prescription drugs, n (%)	7 (41.2%)	21 (47.7)	8 (66.7)	0.379
Multimorbidity, n (%)	13 (76.5)	33 (75.0)	11 (91.7)	0.457
ADL disability, n (%)	**0 (0.0)**	**3 (6.8)**	**3 (25.0)**	**0.047**
IADL disability, n (%)	**1 (5.9)**	**14 (31.8)**	**9 (75.0)**	**<0.001**
Social frailty score (range: 0–5)	**1.00 ± 0.61**	**1.45 ± 1.11**	2.08 ± 1.00^*^	**0.020**
Mini-cognition score (range: 0–5)	**4.41 ± 0.71**	3.61 ± 1.22^*^	3.27 ± 1.27^*^	**0.019**
Cognitive dysfunction, n (%)	**0 (0.0)**	**3 (6.8)**	**3 (25.0)**	**0.047**
PHQ-2 score (range: 0–6)	**0.94 ± 0.83**	**1.48 ± 1.99**	3.82 ± 1.72^*,†^	**<0.001**
Depression, n (%)	**0 (0.0)**	**5 (11.4)**	**8 (66.7)**	**<0.001**

Phenotypic frailty is defined based on the Fried’s criteria. Values are presented as the mean ± standard deviation unless otherwise specified.**Bold** indicates statistically significant values. The comparisons between the three groups were investigated using the analysis of variance with posthoc analysis via Tukey’s honest significance test for continuous variables and Fisher’s exact tests for categorical variables. ^*^ and ^**†**^ indicate statistically significant differences from robust and prefrail groups, respectively.

Abbreviations: ADL, activities of daily living; IADL, instrumental activities of daily living; PHQ-2, patient health questionnaire-2; SPPB, short physical performance battery.

### Difference in serum kynurenine and tryptophan levels and their ratio according to phenotypic fragility status

**Figure 1:**
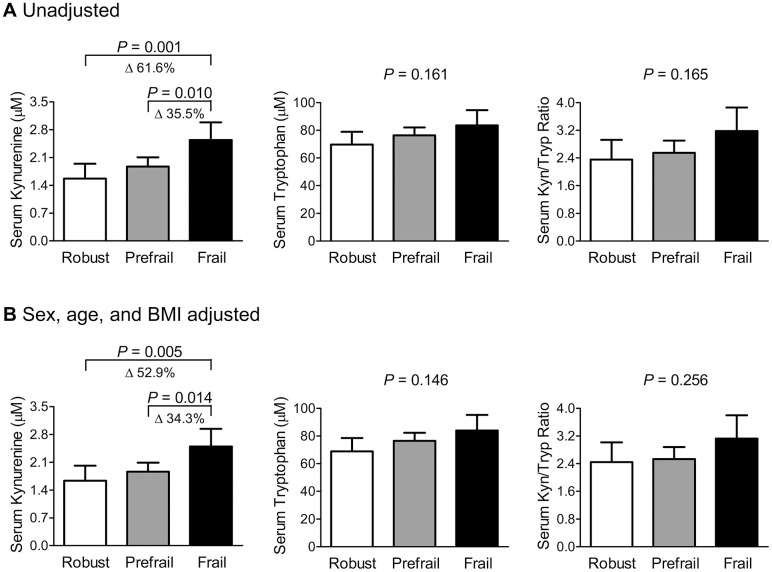
Differences in serum kynurenine and tryptophan levels and their ratio according to the phenotypic frailty status (**A**) before and (**B**) after adjusting for sex, age, and BMI. Phenotypic frailty is defined based on the Fried’s criteria. The estimated means with 95% confidence intervals were generated and compared using an analysis of covariance. Delta (Δ) indicates a change in the value of a variable between groups. Abbreviations: BMI, body mass index; Kyn, kynurenine; Tryp, tryptophan.

Before their levels were adjusted for sex, age, and BMI, the frail group had 61.6% and 35.5% higher serum kynurenine levels than the robust and prefrail groups, respectively (Figure 1A), and the statistical significance persisted after adjusting for these factors (Figure 1B). However, there were no significant differences in serum tryptophan levels and kynurenine/tryptophan ratio between the three groups, regardless of the adjustment models.

### Association between frailty-related parameters and serum kynurenine and tryptophan levels and their ratio

Univariate linear regression analyses showed that serum kynurenine levels and the kynurenine/tryptophan ratio were positively associated with the Rockwood frailty index [17, 18], time to complete 5 chair stands, and PHQ-2 score, and inversely associated with grip strength and gait speed (Table 2). After adjusting for sex, age, and BMI, these correlations were still statistically significant, except for the association of serum kynurenine/|tryptophan ratio with grip strength and PHQ2-score, which showed marginal significance (*P* = 0.061 and 0.062, respectively). However, serum tryptophan levels were not associated with any frailty-related parameters before and after adjusting for potential confounders.

**Table 2:** Linear regression analysis to determine the association of frailty-related factors with serum kynurenine and tryptophan levels and their ratio.

**Unadjusted**	**Serum kynurenine level**					**Serum tryptophan level**					**Serum KTR**			
	**β**	**SE**	***β***	***P***		**β**	**SE**	***β***	***P***		**β**	**SE**	***β***	***P***
Frailty index	**0.055**	**0.010**	**0.546**	**<0.001**		0.001	0.001	0.068	0.565		**0.036**	**0.007**	**0.519**	**<0.001**
Grip strength	**–3.646**	**1.239**	**–0.332**	**0.004**		–0.048	0.056	–0.103	0.387		**–1.985**	**0.870**	**–0.263**	**0.026**
Gait speed	**–0.139**	**0.043**	**–0.361**	**0.002**		–0.002	0.002	–0.101	0.404		**–0.077**	**0.030**	**–0.291**	**0.014**
Chair stand	**2.695**	**1.037**	**0.299**	**0.011**		–0.059	0.046	–0.152	0.206		**2.649**	**0.674**	**0.428**	**<0.001**
PHQ-2 score	**0.833**	**0.266**	**0.350**	**0.003**		0.008	0.012	0.081	0.497		**0.434**	**0.188**	**0.266**	**0.024**
**Sex, age, and BMI adjusted**	**Serum kynurenine level**					**Serum tryptophan level**					**Serum KTR**			
	**β**	**SE**	***β***	***P***		**β**	**SE**	***β***	***P***		**β**	**SE**	***β***	***P***
Frailty index	**0.050**	**0.010**	**0.495**	**<0.001**		0.001	0.001	0.077	0.500		**0.032**	**0.007**	**0.464**	**<0.001**
Grip strength	**–2.264**	**0.818**	**–0.206**	**0.007**		–0.064	0.034	–0.137	0.065		–1.092	0.574	–0.145	0.061
Gait speed	**–0.119**	**0.043**	**–0.310**	**0.008**		–0.002	0.002	–0.115	0.311		**–0.061**	**0.030**	**–0.232**	**0.045**
Chair stand	**2.250**	**1.086**	**0.249**	**0.042**		–0.057	0.045	–0.148	0.210		**2.417**	**0.698**	**0.390**	**0.001**
PHQ-2 score	**0.730**	**0.272**	**0.307**	**0.009**		0.010	0.012	0.097	0.401		0.362	0.190	0.222	0.062

Frailty index is calculated based on the Rockwood’s proposal. The Enter method was applied to this model. **Bold** indicates are statistically significant values. Abbreviations: β, unstandardized regression coefficient; SE, standard error; *β*, standardized regression coefficient; BMI, body mass index; KTR, kynurenine/tryptophan ratio; PHQ-2, patient health questionnaire-2.

### Risk of phenotypic frailty according to the increase in serum kynurenine and tryptophan levels and their ratio

Before and after adjusting for sex, age, and BMI, the odds ratios (ORs) per serum kynurenine increment for phenotypic frailty were approximately 2.6 (Table 3). In contrast, the risk of frailty did not differ according to serum tryptophan concentration and kynurenine/tryptophan ratio in any adjustment model.

**Table 3:** Logistic regression analyses to determine the odds ratios for phenotypic frailty according to the increase in serum kynurenine and tryptophan levels and their ratio.

**Adjustment**	**OR (95% CIs) per serum kynurenine increment**	***P***	**OR (95% CIs) per serum tryptophan increment**	***P***	**OR (95% CIs) per serum KTR increment**	***P***
Unadjusted	**2.67 (1.29–5.52)**	**0.008**	1.02 (0.98–1.06)	0.145	1.52 (0.95–2.42)	0.081
Sex, age, and BMI adjusted	**2.62 (1.22–5.65)**	**0.014**	1.03 (0.99–1.06)	0.119	1.52 (0.93–2.50)	0.098

Phenotypic frailty is defined based on the Fried’s criteria. **Bold** indicates statistically significant values. Abbreviations: BMI; body mass index; CI, confidence interval; KTR, kynurenine/tryptophan ratio; OR, odds ratio.

### Differences in frailty-related parameters according to serum kynurenine tertiles

**Figure 2:**
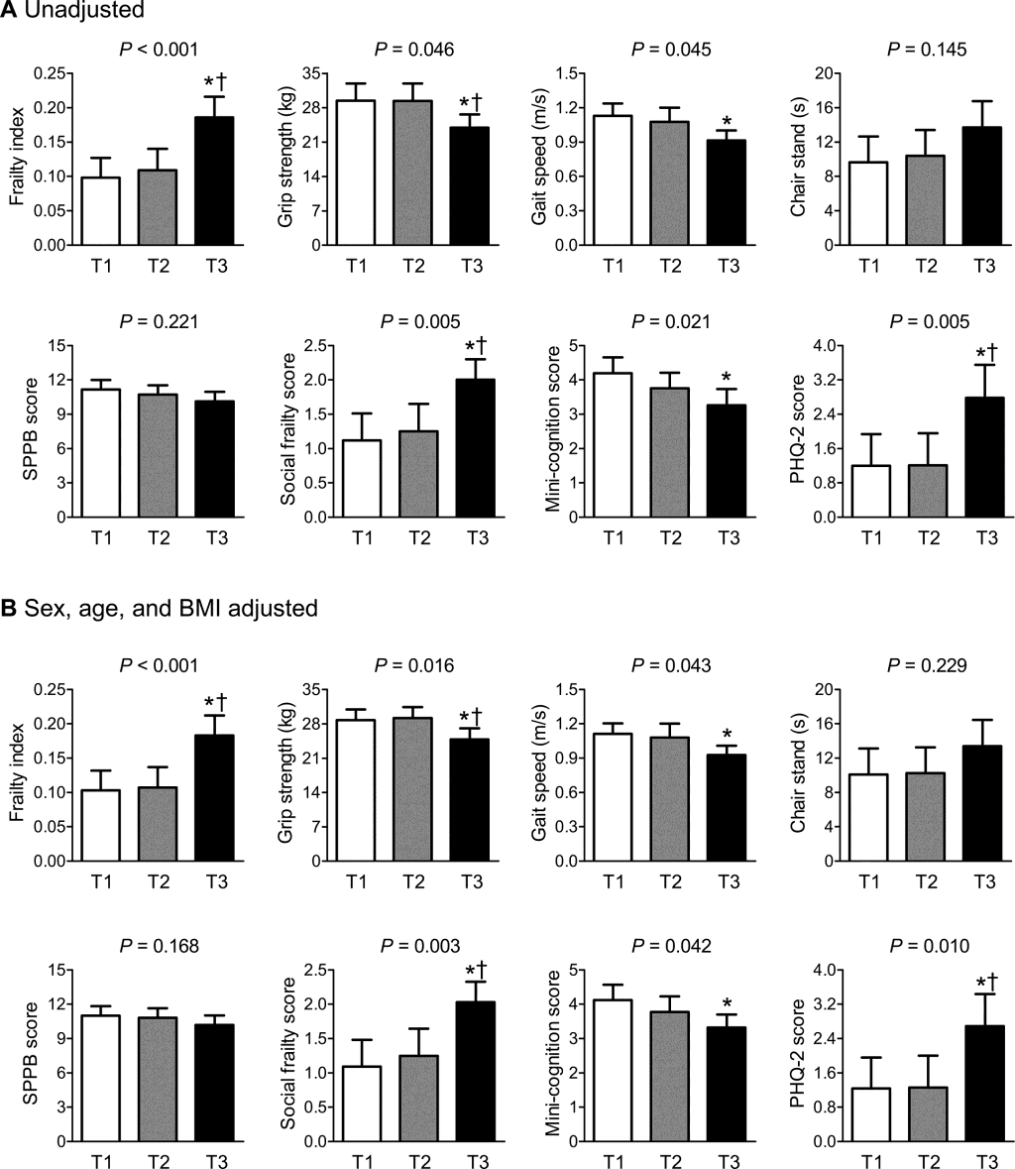
Differences in frailty-related factors according to serum kynurenine tertiles (**A**) before and (**B**) after adjusting for sex, age, and BMI. Frailty index is calculated based on the Rockwood’s proposal. The estimated means with 95% confidence intervals were generated and compared using analysis of covariance. Serum kynurenine tertiles: T1 = 0.78–1.52 μM, T2 = 1.53–2.01 μM, and T3 = 2.02–4.44 μM. ^*^ and ^†^ indicate statistically significant differences from T1 and T2 tertiles, respectively. Abbreviations: PHQ-2, patient health questionnaire-2; SPPB, short physical performance battery.

Among the serum kynurenine and tryptophan levels and their ratio, we specifically focused on serum kynurenine, because this factor was most strongly and consistently related to frailty, as shown above. To investigate whether the association between serum kynurenine levels and frailty-related parameters involved a threshold effect, we divided all the participants into three groups according to their serum kynurenine levels (Figure 2). Compared with those in the lowest kynurenine tertile (T1, 0.78–1.52 μM), the participants with the highest tertile (T3, 2.02–4.44 μM) had a higher frailty index, social frailty score, and PHQ-2 score and lower grip strength, gait speed, and mini-cognition score, before and after adjusting for sex, age, and BMI. In contrast, there were no significant differences in the time to complete 5 chair stands and SPPB score according to serum kynurenine tertiles, regardless of the adjustment models.

### Risk of phenotypic frailty according to serum kynurenine tertiles

**Figure 3:**
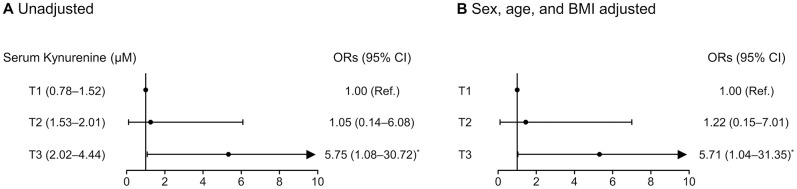
Logistic regression analyses to determine the odds ratios for phenotypic frailty according to serum kynurenine tertiles (**A**) before and (**B**) after adjusting for sex, age, and BMI. Phenotypic frailty is defined based on the Fried’s criteria. ^*^Statistically significant difference from the lowest tertile (T1). Abbreviations: BMI, body mass index; OR, odds ratio; CI, confidence interval.

Logistic regression analyses revealed that the ORs for phenotypic frailty were 5.75-fold and 5.71-fold higher for the participants in the highest kynurenine tertile (T3) than those in the lowest tertile (T1), before and after adjusting for sex, age, and BMI, respectively (Figure 3).

## DISCUSSION

Previous studies have demonstrated a positive association of circulating kynurenine levels with age, fragility hip fracture, and overall mortality. Our new findings here indicate that serum kynurenine levels and the kynurenine/tryptophan ratio were positively associated with the frailty index and inversely associated with grip strength and gait speed. Likewise, the risk of phenotypic frailty was significantly associated with kynurenine levels, before and after adjusting for sex, age, and BMI, but did not differ according to serum tryptophan levels and the kynurenine/tryptophan ratio. These data suggest that kynurenine itself, more so than either tryptophan levels or the kynurenine/tryptophan ratio, is a significant predictor of frailty and overall functional performance. This interpretation is consistent with a previous study that demonstrated that kynurenine treatment decreases muscle contractile strength and muscle fiber size in younger animals, whereas blocking kynurenine production with a tryptophan mimetic can preserve muscle function in older animals [14].

There are several potential mechanisms underlying the observations in our study. Kynurenine is thought to increase oxidative stress [14, 19, 20], and oxidative stress is in turn believed to contribute directly to neuromuscular junction degradation [21]. In addition, kynurenine can cross the blood-brain barrier and has been implicated in cognitive decline and Alzheimer’s disease [22–24]. Recently, it has been proposed that grip strength is an important measure of neurological function [25]. Thus, the association we documented between serum kynurenine levels and grip strength could emanate from the peripheral effects of kynurenine on oxidative stress at the neuromuscular junction and, perhaps centrally, through kynurenine’s effects on the brain.

Frailty is a common clinical syndrome in older adults characterized by a reduced physiological reserve and resistance vulnerable to external stressors [26]. Importantly, a recent longitudinal study demonstrated that frailty is the most promising indicator for biological age among nine different approaches over a 20-year period [27, 28], and this condition is known to lead to poor health outcomes including greater disability, hospitalization, and even death [29]. There is therefore growing interest in potential biomarkers that can detect high-risk individuals early before frailty fully develops [30, 31]. KP metabolites in the blood have been suggested as candidate biomarkers for this purpose by clinical studies that have demonstrated the significant role of tryptophan derivatives on various age-associated degenerative diseases [31]. In fact, two recent human studies have found a correlation between circulating kynurenine/tryptophan ratio and frailty in older Europeans [32, 33]. These studies were meaningful as they were among the first to implicate KP to frailty. However, these studies adopted only the concept proposed by Fried et al. [16], i.e., “phenotypic or physical frailty,” but not “frailty index” proposed by Rockwood and colleagues [17, 18]. The frailty index approach views frailty as a spectrum of aging [34, 35] and is known to be a better predictor of death than physical frailty [36, 37]; therefore, the two studies, though important, may not be enough to fully understand the effects of KP on age-associated frailty. In contrast, we used both operational definitions of frailty, which have been well validated and are widely accepted in aging research [26]; additionally, we included diverse related parameters (such as grip strength, gait speed, time to complete 5 chair stands, SPPB score, social frailty score, mini-cognitive score, and PHQ-2 score), which helped improve the reliability of our results. Furthermore, this was the first clinical study on this topic to be conducted among Asians. We believe that the studies discussed above, including ours, provide an important background for future prospective studies to confirm the role of circulating KP metabolites as a clinical biomarker of frailty.

Several issues and alternatives should be considered when interpreting our data. The first is that kynurenine might be a by-product of inflammation and that although we observed strong associations between kynurenine and measures such as gait speed and grip strength, ultimately these associations might be driven by increased inflammatory cytokine levels. This is certainly possible since previous work has shown that kynurenine levels are significantly correlated with IL-6 levels in older adults [38]. It is also possible that the increase in inflammation, and subsequent rise in kynurenine, is associated with declines in serotonin and melatonin. Presumably, a shift in tryptophan degradation toward the KP will decrease bioavailable serotonin and melatonin, which is likely given the well-established finding that melatonin levels decline with age. The potential decrease in melatonin is particularly relevant for understanding the etiology of frailty, given that melatonin has recently been shown to suppress factors involved in sarcopenia [39] and to inhibit bone loss [40, 41]. Historically, research on kynurenine synthesis has employed the kynurenine/tryptophan ratio as an important measure of kynurenine bioavailability. Future studies could examine the melatonin/kynurenine ratio, given the many important biological functions of melatonin.

There are several potential limitations of this study. Most importantly, the cross-sectional study design precludes any causal inferences about the relationship between serum kynurenine level and frailty. Second, based on previous animal studies that have contributed to muscle health [14, 42], a key phenotype of frailty, we mainly focused on the kynurenine levels; however, other tryptophan derivatives, such as kynurenic acid or quinolinic acid, may also be biologically active and thus implicated in the aging process. Third, the average age of the participants in this study (69.4 years) was considered relatively young for aging research. Therefore, our results may not be entirely applicable to a super-aged population aged >80 years. Lastly, we cannot exclude the possibility that any biased information or uncontrolled factors that affect kynurenine and frailty would interfere the conclusion.

In conclusion, we have demonstrated that serum kynurenine levels were markedly higher in participants with phenotypic frailty than in those without this condition and were positively correlated with the frailty index in older adults. These data are consistent with the results from *in vitro* and animal experiments showing the musculoskeletal weakness and progressive neurodegeneration resulting from kynurenine treatment [9, 13, 14] and provide clinical evidence that circulating kynurenine might be one of attractive biomarkers for assessing frailty risk in humans.

## MATERIALS AND METHODS

### Study participants

The study population consisted of South Koreans who visited the Division of Geriatrics of the Department of Internal Medicine of the Asan Medical Center (Seoul, South Korea) to undergo a comprehensive geriatric assessment between July 2019 and February 2020. We excluded patients with a life expectancy of less than one year due to malignancy, symptomatic heart failure, or end-stage renal failure. We then collected blood samples from the 73 participants who granted their written informed consent for inclusion in this study, which was approved by the Asan Medical Center review board (no. 2020-0259).

### Comprehensive geriatric assessment

Experienced nurses administered a comprehensive geriatric assessment of all participants. Information on demographic characteristics and medical or surgical histories was collected through detailed interviews and reviews of medical records. The protocol for the comprehensive geriatric assessment encompassed comorbidities, functional status, nutritional status, and common geriatric syndromes such as cognitive dysfunction, depression, and polypharmacy.

Multimorbidity was defined as having two or more of the 18 physician-diagnosed conditions including angina, atrial fibrillation/flutter, coronary artery disease, diabetes, heart failure, hypertension, myocardial infarction, peripheral vascular disease, stroke, anxiety disorder, arthritis, asthma, cancer within 5 years, chronic kidney disease (estimated glomerular filtration rate < 60), chronic obstructive lung disease, degenerative spine disease, depression, and sensory impairment. Disability was defined as requiring assistance from another person to perform any of 7 ADLs (feeding, dressing, grooming, walking, getting in and out of bed, toileting, and bathing or showering) or 7 IADLs (making telephone calls, using transportation, shopping, cooking, performing housework, taking medications, and managing money). To assess the participants’ social frailty, we administered the 5-item social frailty questionnaire: 1) going out less frequently; 2) rarely visiting the homes of friends; 3) feeling unhelpful to friends and family; 4) being alone; and 5) not talking with someone every day [43]. Cognitive dysfunction was defined as a score of <24 points on the mini-mental status examination by selected participants identified as positive in the mini-cognition screening test [44]. The selected participants identified as positive in the PHQ-2 screening test were considered to have depression when they scored 10 or more on the 15-item Korean version of the short form of the Geriatric Depression Scale (SGDS-K) [45].

### Functional status assessment

Handgrip strength of the dominant side was measured using a Jamar hydraulic hand dynamometer (Patterson Medical, Warrenville, IL, USA) [46]. Participants were instructed to sit comfortably, bend the elbow at 90 degrees, and grip the dynamometer as strong as possible. The maximum value was selected after all tests were conducted twice at intervals of 1 min or more. We measured the participants’ typical gait speed (m/s) for a 4-m walk and the time to complete 5 chair stands (s) [47]. The SPPB consists of repeated chair stands, standing balance, and gait speed [48]. In the standing balance test, including the side-by-side stance, semi-tandem stance, and tandem stance, the participants were instructed to stand for up to 10 seconds. The score ranged from 0 to 12 points, with a higher SPPB score indicating better leg function.

### Frailty assessment

1) Phenotypic frailty: We evaluated frailty according to the Cardiovascular Health Study frailty criteria, a widely validated definition for frailty, proposed by Fried et al. [16]. The frailty phenotype scale is calculated by assigning a point to the following five components that are relevant to a given individual: self-reported exhaustion, low physical activity, weakness, slowness, and unintentional weight loss. The method employed in our study for conducting these assessments has been previously described [49]. Based on the total score, the participants were classified as robust (0 points), prefrail (1–2 points), or frail (3–5 points).

2) Deficit-accumulation frailty index: The frailty index, proposed by Rockwood et al., is known as the most sensitive predictor of adverse health outcomes and is based on the cumulative effect of medical, functional, and psychosocial age-related deficits [17, 18]. In this study, we calculated a frailty index that has been validated in other studies (see the complete list of assessed items in the Supplementary Material) [26, 50]. The ratio between the number of identified deficits and 50 evaluable items is calculated from 0 to 1, with higher frailty index values indicating a higher frailty status.

### Measurement of kynurenine and tryptophan in human serum

Blood samples were collected from the antecubital vein of each participant when they were at rest in the morning after having fasted overnight for at least 8 hours. After centrifuging the samples at 3000 rpm for 5 min at 4° C, we carefully collected the supernatants to exclude cell components and discarded all samples with hemolysis or clotting. We mixed 50-μL human serum with 200-μL chloroform/methanol (1/2, v/v) and then added an internal standard solution containing 0.6-μM tryptophan-d_5_ (Sigma-Aldrich, St. Louis, MO, USA). The sample was centrifuged at 14,000 rpm for 15 min. We then collected the supernatant, added 100 μL each of H_2_O and chloroform, vigorously mixed the sample and centrifuged it at 4000 rpm for 20 min. We employed the aqueous phase for chemical derivatization using phenyl isothiocyanate. After the reaction, we extracted the derivatization products with 5-mM ammonium acetate in methanol, which were ready for liquid chromatography-tandem mass spectrometry (LC-MS/MS) analysis.

We measured the kynurenine and tryptophan levels by LC-MS/MS using a 1290 high performance liquid chromatography system (Agilent, Waldbronn, Germany), QTRAP 5500 system (AB Sciex, Toronto, Canada), and a reverse phase column (Zorbax Eclipse XDB-C18, 100×2.0 mm). We injected 3 μL into the LC-MS/MS system and ionized it with a turbo spray ionization source. We employed 0.2% formic acid in H_2_O and 0.2% formic acid in acetonitrile as mobile phases A and B, respectively. The separation gradient was as follows: hold at 0% B for 0.5 min, 0 to 95% B for 5 min, 95% B for 1 min, and 95 to 0% B for 0.5 min, then hold at 0% B for 2.5 min. The LC flow was 500 μL/min, and the column temperature was kept at 50° C. We employed multiple reaction monitoring in the positive ion mode and used the extracted ion chromatogram corresponding to the specific transition for each amino acid for quantitation. The calibration range was generally from 1 nM to 600 μM with R^2^ > 0.98. Data analysis was performed using Analyst 1.5.2 software.

### Statistical analysis

All data are presented as means ± standard deviation or as numbers and percentages unless otherwise specified. The baseline characteristics of the study participants according to phenotypic frailty status were compared using an analysis of variance with posthoc analysis via Tukey’s honest significance test for continuous variables and Fisher’s exact tests for categorical variables. Using an analysis of covariance, we generated and compared the estimated means with 95% confidence intervals for the serum kynurenine and tryptophan levels and their ratio according to the phenotypic frailty status and for the frailty-related factors according to serum kynurenine tertiles, before and after adjusting for sex, age, and BMI. We investigated the association between the frailty-related factors and the serum kynurenine and tryptophan levels and their ratio using a linear regression analysis. To generate the ORs for phenotypic frailty according to the increase in serum kynurenine and tryptophan levels and their ratio and according to serum kynurenine tertiles, we performed a logistic regression analysis. All statistical analyses were performed using SPSS, version 18.0 (SPSS Inc., Chicago, IL, USA). We considered *P* < 0.05 to indicate statistical significance.

## Supplementary Material

Supplementary Table 1

## References

[1] Baumgartner RN, Koehler KM, Gallagher D, Romero L, Heymsfield SB, Ross RR, Garry PJ, Lindeman RD. Epidemiology of sarcopenia among the elderly in New Mexico. Am J Epidemiol. 1998; 147:755–63. 10.1093/oxfordjournals.aje.a009520 9554417

[2] Boyle PA, Buchman AS, Wilson RS, Leurgans SE, Bennett DA. Association of muscle strength with the risk of Alzheimer disease and the rate of cognitive decline in community-dwelling older persons. Arch Neurol. 2009; 66:1339–44. 10.1001/archneurol.2009.240 19901164 PMC2838435

[3] Burns JM, Johnson DK, Watts A, Swerdlow RH, Brooks WM. Reduced lean mass in early Alzheimer disease and its association with brain atrophy. Arch Neurol. 2010; 67:428–33. 10.1001/archneurol.2010.38 20385908 PMC2855150

[4] Demontis F, Piccirillo R, Goldberg AL, Perrimon N. The influence of skeletal muscle on systemic aging and lifespan. Aging Cell. 2013; 12:943–49. 10.1111/acel.12126 23802635 PMC3838468

[5] Gray SL, Anderson ML, Hubbard RA, LaCroix A, Crane PK, McCormick W, Bowen JD, McCurry SM, Larson EB. Frailty and incident dementia. J Gerontol A Biol Sci Med Sci. 2013; 68:1083–90. 10.1093/gerona/glt013 23419778 PMC3738027

[6] Nourhashémi F, Andrieu S, Gillette-Guyonnet S, Reynish E, Albarède JL, Grandjean H, Vellas B. Is there a relationship between fat-free soft tissue mass and low cognitive function? results from a study of 7,105 women. J Am Geriatr Soc. 2002; 50:1796–801. 10.1046/j.1532-5415.2002.50507.x 12410897

[7] Kim G, Kim JH. Impact of skeletal muscle mass on metabolic health. Endocrinol Metab (Seoul). 2020; 35:1–6. 10.3803/EnM.2020.35.1.1 32207258 PMC7090295

[8] Cervenka I, Agudelo LZ, Ruas JL. Kynurenines: tryptophan’s metabolites in exercise, inflammation, and mental health. Science. 2017; 357:eaaf9794. 10.1126/science.aaf9794 28751584

[9] Hamrick MW, Isales CM. Special issue: the kynurenine pathway in aging. Exp Gerontol. 2020; 134:110895. 10.1016/j.exger.2020.110895 32151793

[10] Pertovaara M, Raitala A, Lehtimäki T, Karhunen PJ, Oja SS, Jylhä M, Hervonen A, Hurme M. Indoleamine 2,3-dioxygenase activity in nonagenarians is markedly increased and predicts mortality. Mech Ageing Dev. 2006; 127:497–99. 10.1016/j.mad.2006.01.020 16513157

[11] van der Goot AT, Zhu W, Vázquez-Manrique RP, Seinstra RI, Dettmer K, Michels H, Farina F, Krijnen J, Melki R, Buijsman RC, Ruiz Silva M, Thijssen KL, Kema IP, et al. Delaying aging and the aging-associated decline in protein homeostasis by inhibition of tryptophan degradation. Proc Natl Acad Sci USA. 2012; 109:14912–17. 10.1073/pnas.1203083109 22927396 PMC3443121

[12] Oxenkrug GF, Navrotskaya V, Voroboyva L, Summergrad P. Extension of life span of drosophila melanogaster by the inhibitors of tryptophan-kynurenine metabolism. Fly (Austin). 2011; 5:307–09. 10.4161/fly.5.4.18414 22041575 PMC3266072

[13] Refaey ME, McGee-Lawrence ME, Fulzele S, Kennedy EJ, Bollag WB, Elsalanty M, Zhong Q, Ding KH, Bendzunas NG, Shi XM, Xu J, Hill WD, Johnson MH, et al. Kynurenine, a tryptophan metabolite that accumulates with age, induces bone loss. J Bone Miner Res. 2017; 32:2182–93. 10.1002/jbmr.3224 28727234 PMC5685888

[14] Kaiser H, Yu K, Pandya C, Mendhe B, Isales CM, McGee-Lawrence ME, Johnson M, Fulzele S, Hamrick MW. Kynurenine, a tryptophan metabolite that increases with age, induces muscle atrophy and lipid peroxidation. Oxid Med Cell Longev. 2019; 2019:9894238. 10.1155/2019/9894238 31737181 PMC6815546

[15] Kim BJ, Hamrick MW, Yoo HJ, Lee SH, Kim SJ, Koh JM, Isales CM. The detrimental effects of kynurenine, a tryptophan metabolite, on human bone metabolism. J Clin Endocrinol Metab. 2019; 104:2334–42. 10.1210/jc.2018-02481 30715395 PMC6497841

[16] Fried LP, Tangen CM, Walston J, Newman AB, Hirsch C, Gottdiener J, Seeman T, Tracy R, Kop WJ, Burke G, McBurnie MA, and Cardiovascular Health Study Collaborative Research Group. Frailty in older adults: evidence for a phenotype. J Gerontol A Biol Sci Med Sci. 2001; 56:M146–56. 10.1093/gerona/56.3.m146 11253156

[17] Rockwood K, Mitnitski A. Frailty in relation to the accumulation of deficits. J Gerontol A Biol Sci Med Sci. 2007; 62:722–27. 10.1093/gerona/62.7.722 17634318

[18] Rockwood K, Song X, MacKnight C, Bergman H, Hogan DB, McDowell I, Mitnitski A. A global clinical measure of fitness and frailty in elderly people. CMAJ. 2005; 173:489–95. 10.1503/cmaj.050051 16129869 PMC1188185

[19] Sas K, Szabó E, Vécsei L. Mitochondria, oxidative stress and the kynurenine system, with a focus on ageing and neuroprotection. Molecules. 2018; 23:191. 10.3390/molecules23010191 29342113 PMC6017505

[20] Kaiser H, Parker E, Hamrick MW. Kynurenine signaling through the aryl hydrocarbon receptor: implications for aging and healthspan. Exp Gerontol. 2020; 130:110797. 10.1016/j.exger.2019.110797 31786316 PMC7899131

[21] Taetzsch T, Valdez G. NMJ maintenance and repair in aging. Curr Opin Physiol. 2018; 4:57–64. 10.1016/j.cophys.2018.05.007 30560223 PMC6294463

[22] Chatterjee P, Goozee K, Lim CK, James I, Shen K, Jacobs KR, Sohrabi HR, Shah T, Asih PR, Dave P, ManYan C, Taddei K, Lovejoy DB, et al. Alterations in serum kynurenine pathway metabolites in individuals with high neocortical amyloid-β load: a pilot study. Sci Rep. 2018; 8:8008. 10.1038/s41598-018-25968-7 29789640 PMC5964182

[23] Giil LM, Midttun Ø, Refsum H, Ulvik A, Advani R, Smith AD, Ueland PM. Kynurenine pathway metabolites in Alzheimer’s disease. J Alzheimers Dis. 2017; 60:495–504. 10.3233/JAD-170485 28869479

[24] Widner B, Leblhuber F, Walli J, Tilz GP, Demel U, Fuchs D. Tryptophan degradation and immune activation in Alzheimer’s disease. J Neural Transm (Vienna). 2000; 107:343–53. 10.1007/s007020050029 10821443

[25] Carson RG. Get a grip: individual variations in grip strength are a marker of brain health. Neurobiol Aging. 2018; 71:189–222. 10.1016/j.neurobiolaging.2018.07.023 30172220

[26] Lee H, Lee E, Jang IY. Frailty and comprehensive geriatric assessment. J Korean Med Sci. 2020; 35:e16. 10.3346/jkms.2020.35.e16 31950775 PMC6970074

[27] Li X, Ploner A, Wang Y, Magnusson PK, Reynolds C, Finkel D, Pedersen NL, Jylhävä J, Hägg S. Longitudinal trajectories, correlations and mortality associations of nine biological ages across 20-years follow-up. Elife. 2020; 9:e51507. 10.7554/eLife.51507 32041686 PMC7012595

[28] Moskalev A. The challenges of estimating biological age. Elife. 2020; 9:e54969. 10.7554/eLife.54969 32041685 PMC7012619

[29] Clegg A, Young J, Iliffe S, Rikkert MO, Rockwood K. Frailty in elderly people. Lancet. 2013; 381:752–62. 10.1016/S0140-6736(12)62167-9 23395245 PMC4098658

[30] Zampino M, Ferrucci L, Semba RD. Biomarkers in the path from cellular senescence to frailty. Exp Gerontol. 2020; 129:110750. 10.1016/j.exger.2019.110750 31678465 PMC12153401

[31] Kim BJ, Lee SH, Koh JM. Clinical insights into the kynurenine pathway in age-related diseases. Exp Gerontol. 2020; 130:110793. 10.1016/j.exger.2019.110793 31765740

[32] Valdiglesias V, Marcos-Pérez D, Lorenzi M, Onder G, Gostner JM, Strasser B, Fuchs D, Bonassi S. Immunological alterations in frail older adults: a cross sectional study. Exp Gerontol. 2018; 112:119–26. 10.1016/j.exger.2018.09.010 30240849

[33] Marcos-Pérez D, Sánchez-Flores M, Maseda A, Lorenzo-López L, Millán-Calenti JC, Strasser B, Gostner JM, Fuchs D, Pásaro E, Valdiglesias V, Laffon B. Frailty status in older adults is related to alterations in indoleamine 2,3-dioxygenase 1 and guanosine triphosphate cyclohydrolase I enzymatic pathways. J Am Med Dir Assoc. 2017; 18:1049–57. 10.1016/j.jamda.2017.06.021 28801236

[34] Won CW. Frailty: Its Scope and Implications for Geriatricians. Ann Geriatr Med Res. 2019; 23:95–97. 10.4235/agmr.19.0032 32743296 PMC7370768

[35] Walston J, Bandeen-Roche K, Buta B, Bergman H, Gill TM, Morley JE, Fried LP, Robinson TN, Afilalo J, Newman AB, López-Otín C, De Cabo R, Theou O, et al. Moving frailty toward clinical practice: NIA intramural frailty science symposium summary. J Am Geriatr Soc. 2019; 67:1559–64. 10.1111/jgs.15928 31045254 PMC6830521

[36] Theou O, Brothers TD, Mitnitski A, Rockwood K. Operationalization of frailty using eight commonly used scales and comparison of their ability to predict all-cause mortality. J Am Geriatr Soc. 2013; 61:1537–51. 10.1111/jgs.12420 24028357

[37] Blodgett J, Theou O, Kirkland S, Andreou P, Rockwood K. Frailty in NHANES: comparing the frailty index and phenotype. Arch Gerontol Geriatr. 2015; 60:464–70. 10.1016/j.archger.2015.01.016 25697060

[38] Lustgarten MS, Fielding RA. Metabolites associated with circulating interleukin-6 in older adults. J Gerontol A Biol Sci Med Sci. 2017; 72:1277–83. 10.1093/gerona/glw039 26975982 PMC6279159

[39] Sayed RK, Fernández-Ortiz M, Diaz-Casado ME, Rusanova I, Rahim I, Escames G, López LC, Mokhtar DM, Acuña-Castroviejo D. The protective effect of melatonin against age-associated, sarcopenia-dependent tubular aggregate formation, lactate depletion, and mitochondrial changes. J Gerontol A Biol Sci Med Sci. 2018; 73:1330–38. 10.1093/gerona/gly059 29562315

[40] Chen W, Chen X, Chen AC, Shi Q, Pan G, Pei M, Yang H, Liu T, He F. Melatonin restores the osteoporosis-impaired osteogenic potential of bone marrow mesenchymal stem cells by preserving SIRT1-mediated intracellular antioxidant properties. Free Radic Biol Med. 2020; 146:92–106. 10.1016/j.freeradbiomed.2019.10.412 31669348 PMC9805353

[41] Ikegame M, Hattori A, Tabata MJ, Kitamura KI, Tabuchi Y, Furusawa Y, Maruyama Y, Yamamoto T, Sekiguchi T, Matsuoka R, Hanmoto T, Ikari T, Endo M, et al. Melatonin is a potential drug for the prevention of bone loss during space flight. J Pineal Res. 2019; 67:e12594. 10.1111/jpi.12594 31286565 PMC6771646

[42] Dukes A, Davis C, El Refaey M, Upadhyay S, Mork S, Arounleut P, Johnson MH, Hill WD, Isales CM, Hamrick MW. The aromatic amino acid tryptophan stimulates skeletal muscle IGF1/p70s6k/mTor signaling in vivo and the expression of myogenic genes in vitro. Nutrition. 2015; 31:1018–24. 10.1016/j.nut.2015.02.011 26059377 PMC4465076

[43] Park H, Jang IY, Lee HY, Jung HW, Lee E, Kim DH. Screening value of social frailty and its association with physical frailty and disability in community-dwelling older Koreans: aging study of PyeongChang rural area. Int J Environ Res Public Health. 2019; 16:2809. 10.3390/ijerph16162809 31394719 PMC6720732

[44] Kim TH, Jhoo JH, Park JH, Kim JL, Ryu SH, Moon SW, Choo IH, Lee DW, Yoon JC, Do YJ, Lee SB, Kim MD, Kim KW. Korean version of mini mental status examination for dementia screening and its’ short form. Psychiatry Investig. 2010; 7:102–08. 10.4306/pi.2010.7.2.102 20577618 PMC2890863

[45] Kroenke K, Spitzer RL, Williams JB. The patient health questionnaire-2: validity of a two-item depression screener. Med Care. 2003; 41:1284–92. 10.1097/01.MLR.0000093487.78664.3C 14583691

[46] Roberts HC, Denison HJ, Martin HJ, Patel HP, Syddall H, Cooper C, Sayer AA. A review of the measurement of grip strength in clinical and epidemiological studies: towards a standardised approach. Age Ageing. 2011; 40:423–29. 10.1093/ageing/afr051 21624928

[47] Peel NM, Kuys SS, Klein K. Gait speed as a measure in geriatric assessment in clinical settings: a systematic review. J Gerontol A Biol Sci Med Sci. 2013; 68:39–46. 10.1093/gerona/gls174 22923430

[48] Jung HW, Roh H, Cho Y, Jeong J, Shin YS, Lim JY, Guralnik JM, Park J. Validation of a multi-sensor-based kiosk for short physical performance battery. J Am Geriatr Soc. 2019; 67:2605–09. 10.1111/jgs.16135 31441514

[49] Won CW, Lee S, Kim J, Chon D, Kim S, Kim CO, Kim MK, Cho B, Choi KM, Roh E, Jang HC, Son SJ, Lee JH, et al. Korean frailty and aging cohort study (KFACS): cohort profile. BMJ Open. 2020; 10:e035573. 10.1136/bmjopen-2019-035573 32327477 PMC7204935

[50] Kim DH, Afilalo J, Shi SM, Popma JJ, Khabbaz KR, Laham RJ, Grodstein F, Guibone K, Lux E, Lipsitz LA. Evaluation of changes in functional status in the year after aortic valve replacement. JAMA Intern Med. 2019; 179:383–91. 10.1001/jamainternmed.2018.6738 30715097 PMC6439710

