# Supplementary material for: The association of circulating kynurenine, a tryptophan metabolite, with frailty in older adults
Source: Aging (Albany NY). 2020 Nov 13;12(21):22253–65. doi: 10.18632/aging.104179 (PMC11623974; doi:10.18632/aging.104179)
Supplement: Supplementary Table 1 [file aging-12-104179-s001.pdf]

Supplementary Table

Supplementary Table 1. Items for comprehensive geriatric assessment-frailty index.

**Medical History (21 items)**		
• Angina	• COPD	• Heart failure
• Anxiety disorder	• Coronary artery disease	• Hypertension
• Arthritis	• Degenerative spine disease	• Myocardial infarction
• Asthma	• Dementia	• Peripheral vascular disease
• Atrial fibrillation/flutter	• Depression	• Sensory impairment
• Cancer within 5 years	• Diabetes	• Stroke/TIA
• Chronic kidney disease (eGFR < 60)	• Fall within the past year	• Use of ≥ 5 prescription drugs
**Functional Status (22 items)**		
**Activities of Daily Living**	**Activities of Daily Living**	**Nagi and Rosow-Breslau Activities**
• Feeding	• Using telephone	• Pulling or pushing a large object
• Dressing/undressing	• Using transportation	• Stooping, crouching or kneeling
• Grooming	• Shopping	• Lifting or carrying 10 lbs
• Walking (or use of a walker)	• Preparing own meals	• Reaching arms above shoulder
• Getting in and out of bed	• Housework	• Writing or handling small objects
• Toileting	• Taking own medications	• Walking up/down a flight of stairs
• Bathing or shower	• Managing money	• Heavy work around house

**Performance Tests (4 items)**	
**Mini-Mental Status Examination**	**5 Repeated Chair Stands**
27–30 points (0 points)	<11.20 s (0 points)
24–26 points (0.3 points)	11.20–13.69 s (0.25 points)
21–23 points (0.7 points)	13.70–16.69 s (0.5 points)
<21 points (1 point)	16.70–60.9 s (0.75 points)
	≥ 61.0 s (1 point)
**Gait Speed**	**Dominant Handgrip Strength**
≥ 1 m/sec (0 points)	M, ≥ 32 kg; F, ≥ 20 kg (0 points)
0.80–0.99 m/s (0.3 points)	M, ≥ 26–31 kg; F, 16–19 kg (0.5 points)
0.60–0.79 m/sec (0.7 points)	M, <26 kg; F, <16 kg (1 point)
<0.60 m/s (1 point)	

**Nutritional Status (3 items)**		
• Weight loss > 4.5 kg in past year	• Body mass index < 21 kg/m^2	• Serum albumin < 3.5 g/dL

Abbreviations: COPD, chronic obstructive pulmonary disease; eGFR, estimated glomerular filtration rate; F, female; M, male; TIA, transient ischemic attack.
